# The Baboon Kidney Transcriptome: Analysis of Transcript Sequence, Splice Variants, and Abundance

**DOI:** 10.1371/journal.pone.0057563

**Published:** 2013-04-23

**Authors:** Kimberly D. Spradling, Jeremy P. Glenn, Roy Garcia, Robert E. Shade, Laura A. Cox

**Affiliations:** 1 Department of Genetics, Texas Biomedical Research Institute, San Antonio, Texas, United States of America; 2 Southwest National Primate Research Center, Texas Biomedical Research Institute, San Antonio, Texas, United States of America; University of California, Los Angeles, United States of America

## Abstract

The baboon is an invaluable model for the study of human health and disease, including many complex diseases of the kidney. Although scientists have made great progress in developing this animal as a model for numerous areas of biomedical research, genomic resources for the baboon, such as a quality annotated genome, are still lacking. To this end, we characterized the baboon kidney transcriptome using high-throughput cDNA sequencing (RNA-Seq) to identify genes, gene variants, single nucleotide polymorphisms (SNPs), insertion-deletion polymorphisms (InDels), cellular functions, and key pathways in the baboon kidney to provide a genomic resource for the baboon. Analysis of our sequencing data revealed 45,499 high-confidence SNPs and 29,813 InDels comparing baboon cDNA sequences with the human hg18 reference assembly and identified 35,900 cDNAs in the baboon kidney, including 35,150 transcripts representing 15,369 genic genes that are novel for the baboon. Gene ontology analysis of our sequencing dataset also identified numerous biological functions and canonical pathways that were significant in the baboon kidney, including a large number of metabolic pathways that support known functions of the kidney. The results presented in this study catalogues the transcribed mRNAs, noncoding RNAs, and hypothetical proteins in the baboon kidney and establishes a genomic resource for scientists using the baboon as an experimental model.

## Introduction

The baboon (*Papio sp.*) is an essential animal model in biomedical research due to its close phylogenetic proximity to humans. The use of baboons for biomedical research in the United States began in April of 1956 when scientists discovered that a 16-year-old female baboon from the New Orleans' Audubon Park Zoo had naturally developed arterial disease similar to that seen in humans [1]. Following this discovery, the baboon quickly gained interest in the scientific community as an experimental animal model for the study of atherosclerosis [2]–[11]. During the last 50 years, the baboon has become a well-characterized and validated primate model for many other areas of biomedical research as well, including cholesterol metabolism [12]–[14], hypertension [15], obesity [16], diabetes [17], [18], embryology [19]–[21], endometriosis [22], epilepsy [23], osteoporosis [24], alcoholic liver disease [25], gastroesophageal reflux disease (GERD) [26], and a variety of infectious diseases [27]–[30].

Although smaller animal models, such as rodents, have provided a large amount of insight into numerous areas of scientific research, the use of nonhuman primates (NHPs) is essential for studying genetic variation underlying complex physiological and disease processes of humans. The baboon has become one of the most popular laboratory primate species and is the most commonly used primate model for the genetic studies of complex traits and susceptibility to complex diseases [31]. The success of the baboon model in biomedical research is attributed to its genetic, physiologic, and anatomic similarity to humans. Directly relevant to the kidney, hypertension studies have shown that systolic blood pressure, diastolic blood pressure, and sodium-lithium countertransport activity (SLC) are highly heritable in baboons and are similar in magnitude to those observed in humans [15], [32], [33]. SLC corresponds to blood pressure response to dietary salt [34]. It is well-established that SLC is heritable in primates but not in rodents, underlying the need for studies in baboon to understand genetic variation underlying variation in blood pressure in humans.

Construction of the genetic linkage map for the baboon genome has been a valuable resource for genetic and genomic studies using this animal model. The first-generation linkage map was published in 2000 [35] and later improved in 2006 by the addition of more loci in chromosomal regions with insufficient marker density in the initial map [36]. This map has allowed scientists to localize and identify functionally significant genes that influence phenotypic variation related to human health or disease [11], [14], [37]–[42].

To further enhance the baboon as a model for biomedical research, we used high-throughput cDNA sequencing (RNA-Seq) to analyze the baboon kidney transcriptome. RNA-Seq is a powerful method for comprehensive transcriptome analysis that employs deep-sequencing technologies for discovering, profiling, and quantifying RNA transcripts [43]–[49]. Since the mid-1990s, microarray analysis has been the main molecular tool used for high-throughput measurement of gene expression levels [50]–[55]. However, RNA-Seq has been shown to offer key advantages over microarrays in measuring gene expression profiles, such as identifying alternative splicing events, single nucleotide polymorphisms (SNPs), insertion-deletion polymorphisms (InDels), allele-specific expression, and rare or novel transcripts. Because RNA-Seq does not require species- and transcript-specific probes, the data are not biased by previous assumptions about the nature of the transcriptome. Therefore, RNA–Seq allows scientists to investigate species with poor or missing genomic annotations, such as the baboon.

The mammalian kidney is a complex organ that is essential for numerous regulatory functions by mechanisms of filtration, reabsorption, and secretion. The kidney plays a critical role in regulating hormonal and homeostatic functions as it produces a variety of hormones (including calcitriol, erythropoietin, and the enzyme renin) and regulates electrolytes, acid-base balance, and blood pressure by maintaining homeostasis of extracellular fluids [56]–[59]. To gain a more comprehensive understanding of these complex biological processes, we used our RNA-Seq dataset to assess gene expression, cellular functions, and key pathways in the baboon kidney. A recent study [43] used RNA-Seq to compare the transcriptome composition across numerous human and mouse cell lines and tissues, including the kidney. The findings of this study revealed that approximately 75% of protein-coding genes are ubiquitously expressed across all tissues and cell lines. Thus, the majority of transcripts identified in our analysis of the kidney are presumed to be ubiquitously expressed genes and provide information on the core transcriptome of the baboon, although these ubiquitous genes can still vary in relative expression levels between tissues and in expression of alternative mRNA isoforms. In addition to ubiquitously expressed genes, our analysis also identified gene expression patterns specialized for the baboon kidney. Therefore, this study provides data on ubiquitously expressed genes that account for the majority of the baboon transcriptome, as well as gene expression specialized for the kidney, and establishes a transcriptome resource for scientists using the baboon as an experimental model.

## Results

### Sample sequencing and read mapping

Kidney biopsy samples were obtained from 14 baboons of different sex and ranging in age from 6 to 23 years. A cDNA library was generated from each collected tissue and sequenced using the Genome Analyzer IIx (GAIIx) system (Illumina, Inc., San Diego, CA). The 101-bp paired-end sequencing runs generated a total of 299,341,739 reads, of which 80.63% mapped to a unique location in the human reference genome (March 2006 (NCBI36/hg18)) when allowing for up to two mismatches per 25-bp read segment, or up to eight total mismatches per 100 bp. FastQC of the 14 individual BAM files and the combined BAM file used for consensus sequence build and SNP calling all passed the quality check for primer contamination, and all but one BAM file passed the per base sequence quality check. The file that failed the per base sequence quality check had the lowest total sequence count (<two million), and bases 95–101 had lower quartile values that fell below a Phred quality score of 10. However, the median Phred score for these bases stayed at 28, which is well above the Phred quality cutoff of 20 that FastQC recommends. The aligned sequencing reads were then used to identify known RefSeq genes annotated in the human hg18 genome assembly as well as novel transcribed regions in the baboon kidney transcriptome.

### Identification of coding transcripts in the baboon kidney

After applying a 1× coverage cutoff, reference analysis of the mapped sequencing reads provided sequence data for 20,714 coding RNA transcripts (Table S1) and transcripts for 1,118 hypothetical proteins or open reading frames (Table S2) annotated in the human hg18 genome assembly that ranged from 1- to 49,042-fold coverage and had expression values that ranged from 1.99×10^4^ to 1.77×10^9^ Fragments Per Kilobase of transcript per Million mapped reads (FPKM). The percentage of n's within the sequence reads ranged from 0 to 95.84%. When aligned with the corresponding human genes, it appeared that most of the n's within the baboon sequences represented exons or untranslated regions (UTRs) that likely differ between the two species. We identified 13,249 genic genes based on common gene identifiers that have been annotated in the human hg18 genome assembly. Of the 13,249 genes identified, 8,888 were represented by only 1 transcript and 4,361 genes were represented by 2–18 transcript variants, with an average of 1.6 variants per gene (Table S3). Chromosomal distribution of the 20,714 annotated coding RNA transcripts is shown in Table 1. The largest number of transcripts and genes identified in the baboon kidney were located on chromosome 1 while the smallest number of transcripts and genes were located on the Y chromosome.

**Table 1:** Chromosomal distribution of 20,714 annotated coding RNA transcripts identified from the reference analysis.

Chr.	Coding Transcripts	Genes with 1 Transcript	Genes with ≥2 Variants	Total Number Genic Genes	Average Variants per Gene
1	2,146	908	457	1,365	1.6
2	1,359	610	277	887	1.5
3	1,265	485	283	768	1.7
4	801	339	167	506	1.6
5	922	429	190	619	1.5
6	1,027	494	200	694	1.5
7	979	414	208	622	1.6
8	722	304	145	449	1.6
9	847	347	177	524	1.6
10	820	320	171	491	1.7
11	1,288	529	275	804	1.6
12	1,089	466	231	697	1.6
13	329	150	72	222	1.5
14	660	275	146	421	1.6
15	666	283	141	424	1.6
16	884	422	181	603	1.5
17	1,176	578	222	800	1.5
18	270	120	55	175	1.5
19	1,296	678	248	926	1.4
20	561	198	139	337	1.7
21	221	73	50	123	1.8
22	513	199	117	316	1.6
X	854	258	205	463	1.8
Y	19	11	3	14	1.4
**Average = **	**863**	**370**	**182**	**552**	**1.6**

### Identification of noncoding RNAs in the baboon kidney

Although Illumina's mRNA-Seq Sample Preparation Kit was used to purify mRNA from each total RNA sample, reference analysis of our dataset provided sequences for 1,492 noncoding RNAs that uniquely mapped to the human hg18 genome assembly (Table S4). This list of noncoding RNAs ranged from 1- to 7,095-fold coverage and had expression values that ranged from 2.86×10^4^ to 2.88×10^8^ FPKM. Included in this list were 24 long intergenic noncoding RNAs (lincRNAs), 8 immature pre-micro RNAs (pre-miRNAs), and 31 small nucleolar RNAs (snoRNAs). We identified 1,197 genic genes based on common gene identifiers that have been annotated in the human hg18 genome assembly. Of the 1,197 genes identified, 1,008 were represented by only 1 transcript and 189 genes were represented by 2–13 transcript variants, with an average of 1.2 variants per gene (Table S5). Chromosomal distribution of the 1,492 annotated noncoding RNA transcripts is shown in Table 2. As with the coding RNAs identified in the dataset, the largest number of noncoding transcripts and genes identified in the baboon kidney were located on chromosome 1 while the smallest number of transcripts and genes were located on the Y chromosome.

**Table 2:** Chromosomal distribution of 1,492 annotated noncoding RNA transcripts identified from the reference analysis.

Chr.	Noncoding Transcripts	Genes with 1 Transcript	Genes with ≥2 Variants	Total Number Genic Genes	Average Variants per Gene
1	149	103	21	124	1.2
2	90	65	10	75	1.2
3	94	62	14	76	1.2
4	48	36	5	41	1.2
5	61	43	8	51	1.2
6	73	44	11	55	1.3
7	100	69	12	81	1.2
8	42	26	6	32	1.3
9	58	38	8	46	1.3
10	73	47	11	58	1.3
11	87	56	9	65	1.3
12	82	51	12	63	1.3
13	21	21	0	21	1.0
14	51	23	8	31	1.7
15	42	32	5	37	1.1
16	59	44	7	51	1.2
17	110	58	17	75	1.5
18	15	13	1	14	1.1
19	71	55	7	62	1.2
20	31	27	1	28	1.1
21	18	14	2	16	1.1
22	45	31	6	37	1.2
X	71	49	8	57	1.3
Y	1	1	0	1	1.0
**Average = **	**62**	**42**	**8**	**50**	**1.2**

### Unique baboon splice variants compared with human hg18 reference genome

Cufflinks Reference Annotation Based Transcript (RABT) assembly method identified known and unique transcripts based on the human hg18 reference genome to complement transcripts identified from the reference analysis. Transcripts identified from this reference-guided assembly included those that were identified using the hg18 reference analysis as well as those that were unique compared to the hg18 genome assembly. After applying a 1× coverage cutoff, there were 5,840 transcripts identical to those identified using the reference analysis. After removing the 5,840 duplicated transcripts, there were 10,130 transcript variants identified using both analysis methods that differed in length and/or chromosomal location and 1,697 transcripts uniquely identified using the RABT assembly method. The 11,827 transcripts identified from the RABT assembly included 10,430 coding RNAs that ranged from 1- to 68,905-fold coverage with expression values that ranged from 3.44×10^4^ to 2.70×10^9^ FPKM (Table S6), 678 hypothetical proteins or open reading frames that ranged from 1- to 1,480-fold coverage with expression values that ranged from 4.04×10^4^ to 6.68×10^7^ FPKM (Table S7), and 719 noncoding RNAs (including 22 lincRNAs, 28 pre-miRNAs, and 9 snoRNAs) that ranged from 1- to 8,310-fold coverage with expression values that ranged from 3.04×10^4^ to 3.42×10^8^ FPKM (Table S8).

Baboon transcripts that did not map to the human hg18 reference genome were annotated based on their genomic coordinates within the latest human genome assembly (February 2009 (GRCh37/hg19)). Genomic coordinates of baboon transcript sequences were translated to the hg19 human genome for comparison of genomic intervals. Aligning the chromosomal position of each translated baboon sequence with the hg19 human genome, we identified 350 coding RNA transcripts that ranged from 1- to 762,193-fold coverage, 6 hypothetical proteins or open reading frames that ranged from 1- to 97,747-fold coverage, and 70 noncoding RNAs (including 1 lincRNA, 3 pre-miRNAs, and 1 snoRNA) that ranged from 1- to 136,846-fold coverage (Table S9). The expression values of these transcripts ranged from 3.08×10^4^ to 3.46×10^10^ FPKM.

Excluding the 5,840 transcripts duplicated from the reference analysis, we identified 6,999 genic genes from the list of coding RNA transcripts and 619 genic genes from the list of noncoding RNAs that are considered to be unique baboon splice variants compared with the human hg18 reference genome. Of the 6,999 coding RNA genes identified, 4,708 were represented by only 1 transcript and 2,291 genes were represented by 2–28 transcript variants, with an average of 1.5 variants per gene (Table S10). Of the 619 noncoding RNA genes identified, 518 were represented by only 1 transcript and 101 genes were represented by 2–20 transcript variants, with an average of 1.3 variants per gene (Table S11).

### Unique baboon splice variants compared with human

Genomic coordinates of baboon transcript sequences that were not annotated in the human hg18 or hg19 genome assemblies were translated and mapped to the chimpanzee (March 2006 (CGSC 2.1/panTro2)), orangutan (July 2007 (WUGSC 2.0.2/ponAbe2)), and rhesus (January 2006 (MGSC Merged 1.0/rheMac2)) genomes. Aligning the chromosomal position of each translated baboon sequence with the other NHP genomes, we identified 27 coding RNAs that ranged from 1- to 1,843-fold coverage with expression values that ranged from 3.75×10^4^ to 9.71×10^7^ FPKM and 2 hypothetical proteins (*LOC100499547* and *LOC732516*) (Table S12). Sixteen transcripts were annotated using the ponAbe2 assembly, and 13 transcripts were annotated using the rheMac2 assembly. No additional transcripts were annotated using the panTro2 assembly. Baboon transcript sequences that were not annotated in the human or NHP genome assemblies were then translated and mapped to the rat (November 2004 (Baylor 3.4/rn4)) genome based on genomic interval overlap, using 1 bp as the minimum required overlap between the gene tracks. Aligning the chromosomal position of each translated baboon sequence with the rat genome, we identified 292 coding transcripts that ranged from 2- to 883,506-fold coverage with expression values that ranged from 7.61×10^4^ to 4.20×10^10^ FPKM and 2 hypothetical proteins (*LOC100174909* and *LOC498145*) (Table S13).

Altogether we found 213 genic genes for coding RNA transcripts that were considered to be unique baboon splice variants compared with the human genome. Of the 213 coding RNA genes identified, 146 were represented by only 1 transcript and 67 genes were represented by 2–7 transcript variants, with an average of 1.5 variants per gene (Table S14).

### Comparison of GenBank Papio Genes with Baboon Kidney RNA-Seq Genes

In total, our RNA-Seq dataset of the baboon kidney included 35,900 transcripts, which represented 15,669 genic genes. Of the 15,669 genes identified, 6,124 were represented by only 1 transcript and 9,545 genes were represented by 2–61 transcript variants, with an average of 2.3 variants per gene (Table S15).

GenBank contains 463 RefSeq gene records for baboon (all identified from *Papio anubis*). Comparison of the transcripts identified from sequencing the 14 baboon kidney RNA samples with the GenBank cDNAs revealed the presence of 301 genes in our dataset that had already been identified in baboon. Of the 301 genes identified, 108 were represented by 1 variant and 193 were represented by 2 to 15 variants, with a sum of 750 transcript variants and an average of 2.5 variants per gene (Table S15). Alignment of 108 transcript sequences from our dataset with the corresponding baboon cDNA sequences from GenBank revealed a 7.93% average difference within the coding regions. Most notably were additional 5′- and 3′-UTR nucleotides in the baboon kidney cDNAs that were not included in the *Papio anubis* RefSeq cDNAs. Many stretches of n's in the baboon kidney cDNA sequences didn't align with baboon cDNA sequences from GenBank, which is likely due to the presence of exons in the human genome assembly but not the baboon.

Excluding the 750 variants that represented 301 genes already identified in *Papio anubis*, our dataset included 35,150 transcripts that are novel for the baboon. These 35,150 transcripts represented 15,369 genic genes based on common gene identifiers. The 15,369 genes included 6,018 genes represented by 1 variant and 9,351 genes represented by 2 to 61 variants, with an average of 2.3 variants per gene (Table S15).

### Identification of SNPs in the baboon kidney transcriptome

The GATK Unified Genotyper was used to identify 45,499 high-confidence SNPs among the 14 baboon kidney transcriptome samples (Table S16). There were 10,913 polymorphisms in coding mRNAs. These included 2,080 exonic SNPs, of which 1,268 were synonymous and 800 were nonsynomymous; 6,955 intronic SNPs; 69 SNPs in the 5'UTR; and 1,809 SNPs in the 3'UTR. There were 1,760 polymorphisms in noncoding RNAs, including 1,206 SNPs in noncoding exons and 554 SNPs in noncoding introns. The remaining 32,826 SNPs were located in intergenic regions. Chromosomal distribution of the coding and noncoding transcript SNPs is shown in Tables 3 and 4, respectively. We calculated an average of 455 coding transcript SNPs per chromosome, which indicated the presence of 0.54 SNP per coding transcript and 0.0001558 SNP per coding RNA nucleotide. The average number of noncoding transcript SNPs per chromosome was 73, which indicated the presence of 1.26 SNPs per noncoding transcript and 0.0004864 SNP per coding RNA nucleotide.

**Table 3:** Chromosomal distribution of 10,913 coding transcript SNPs identified among the 14 baboon kidney transcriptome samples.

Chr.	Coding Transcripts	Coding Transcript SNPs	SNPs per Coding Transcript	Coding Transcript Average Length	SNPs per Coding RNA Nucleotide
1	2,146	1,092	0.51	3,323.7	0.0001531
2	1,359	797	0.59	3,649.3	0.0001607
3	1,265	641	0.51	3,717.2	0.0001363
4	801	486	0.61	3,769.5	0.0001610
5	922	452	0.49	3,749.9	0.0001307
6	1,027	720	0.70	3,477.4	0.0002016
7	979	507	0.52	3,381.6	0.0001531
8	722	276	0.38	3,523.1	0.0001085
9	847	478	0.56	3,657.6	0.0001543
10	820	447	0.55	3,555.0	0.0001533
11	1,288	727	0.56	3,142.6	0.0001796
12	1,089	499	0.46	3,541.9	0.0001294
13	329	210	0.64	3,852.9	0.0001657
14	660	325	0.49	3,388.2	0.0001453
15	666	487	0.73	3,881.8	0.0001884
16	884	410	0.46	3,147.6	0.0001474
17	1,176	611	0.52	3,144.8	0.0001652
18	270	173	0.64	4,043.5	0.0001585
19	1,296	555	0.43	2,686.0	0.0001594
20	561	315	0.56	3,089.5	0.0001817
21	221	139	0.63	3,547.0	0.0001773
22	513	312	0.61	3,083.4	0.0001972
X	854	242	0.28	3,459.6	0.0000819
Y	19	12	0.63	4,252.1	0.0001485
**Average = **	**863**	**455**	**0.54**	**3,502.7**	**0.0001558**

**Table 4:** Chromosomal distribution of 1,760 noncoding RNA SNPs identified among the 14 baboon kidney transcriptome samples.

Chr.	Noncoding RNAs	Noncoding RNA SNPs		SNPs per Noncoding RNA		Noncoding RNA Average Length		SNPs per Noncoding RNA Nucleotide		
1	149	116		0.78		2,383.0		0.0003267		
2	90	142		1.58		2,331.2		0.0006768		
3	94	124		1.32		2,702.5		0.0004881		
4	48	45		0.94		2,207.4		0.0004247		
5	61	54		0.89		2,932.4		0.0003019		
6	83	125		1.51		2,330.5		0.0006462		
7	100	79		0.79		2,246.0		0.0003517		
8	42	39		0.93		2,807.5		0.0003308		
9	58	96		1.66		3,020.7		0.0005479		
10	73	102		1.40		2,358.4		0.0005925		
11	87	114		1.31		3,239.5		0.0004045		
12	83	71		0.86		2,738.4		0.0003124		
13	21	26		1.24		2,084.1		0.0005941		
14	51	42		0.82		2,967.5		0.0002775		
15	42	71		1.69		2,497.2		0.0006769		
16	59	99		1.68		2,596.3		0.0006463		
17	110	100		0.91		2,128.6		0.0004271		
18	15	14		0.93		2,801.8		0.0003331		
19	71	87		1.23		1,994.4		0.0006144		
20	31	47		1.52		2,155.4		0.0007034		
21	18	38		2.11		2,544.4		0.0008297		
22	45	57		1.27		2,584.5		0.0004901		
X	71	70		0.99		2,462.7		0.0004003		
Y	1	2		2.00		7,217.0		0.0002771		
**Average = **	**63**		**73**		**1.26**		**2,722.1**		**0.0004864**	

### Identification of InDels

We identified 29,813 InDels comparing baboon cDNA sequences with the human hg18 reference assembly. The chromosomal distribution of the InDels is shown in Table 5.

**Table 5:** Chromosomal distribution of 29,813 InDels identified comparing baboon sequences with human hg18 reference assembly.

Chr.	Baboon to Human hg18 InDels
1	3324
2	2227
3	2020
4	1212
5	1627
6	1511
7	1342
8	1040
9	1100
10	1306
11	1635
12	1729
13	598
14	1114
15	1106
16	1031
17	1633
18	460
19	993
20	830
21	281
22	584
X	1093
Y	17

### Pathway analysis of genes expressed in the baboon kidney transcriptome

The top 800 transcripts associated with a canonical pathway in the Ingenuity Pathways Analysis (IPA) Knowledge Base (Ingenuity® Systems, http://www.ingenuity.com) were considered for gene ontology analysis to identify significant biological functions, canonical pathways, and networks of genes in the baboon kidney transcriptome. Analysis of our dataset revealed 74 biological functions (Table S17) and 52 canonical pathways (Table S18) that were significant in the baboon kidney. The top biological functions identified included those associated with diseases or disorders (neurological disease, skeletal and muscular disorders, cancer, gastrointestinal disease, genetic disorder, developmental disorder, metabolic disease), followed by those associated with molecular and cellular functions (amino acid metabolism, small molecule biochemistry, free radical scavenging, cellular compromise) (Table S17). Many of the significant canonical pathways identified from our dataset included those associated with metabolic functions. These included pathways involving amino acid metabolism (valine/leucine/isoleucine degradation, arginine and proline metabolism, tyrosine metabolism, tryptophan metabolism, glutathione metabolism, phenylalanine metabolism, glycine/serine/threonine metabolism, histidine metabolism, lysine degradation, urea cycle and metabolism of amino groups, β-alanine metabolism, selenoamino acid metabolism, methionine metabolism), carbohydrate metabolism (citrate cycle, pyruvate metabolism, glycolysis and gluconeogenesis, propanoate metabolism, butanoate metabolism, glyoxylate and dicarboxylate metabolism, inositol metabolism, ascorbate and aldarate metabolism, pentose and glucuronate interconversions, fructose and mannose metabolism), energy metabolism (oxidative phosphorylation and methane metabolism), lipid metabolism (fatty acid metabolism and bile acid biosynthesis), metabolism of cofactors and vitamins (ubiquinone biosynthesis, stilbene/coumarine/lignin biosynthesis, folate biosynthesis, metabolism of xenobiotics by cytochrome P450), and metabolism of complex lipids (glycerolipid metabolism) (Table S18). In addition to these metabolic pathways, a number of signaling pathways were identified from the top 800 transcripts in our dataset. The significant signaling pathways identified included those involved in apoptosis, cell cycle regulation, nuclear receptor signaling, and xenobiotic metabolism (aryl hydrocarbon receptor signaling), cancer (polyamine regulation in colon cancer and phosphoinositide 3-kinase (PI3K)/protein kinase B (AKT) signaling), cardiovascular signaling (intrinsic prothrombin activation pathway), cellular growth/proliferation/development (eukaryotic initiation factor 2 (EIF2) signaling, regulation of EIF4 and p70 ribosomal 56 protein kinase (p7056K) signaling, PI3K/AKT signaling, mammalian target of rapamycin (mTOR) signaling), cellular immune response (antigen presentation pathway, clathrin-mediated endocytosis signaling, lipid antigen presentation by cluster of differentiation 1 (CD1), OX40 signaling pathway), cellular stress and injury (EIF2 signaling, regulation of EIF4 and p7056K signaling, nuclear factor (erythroid-derived 2)-like 2 (NRF2)-mediated oxidative stress response, intrinsic prothrombin activation pathway), disease-specific pathways (mitochondrial dysfunction, polyamine regulation in colon cancer, neuroprotective role of thimet oligopeptidase (THOP1) in Alzheimer's disease), humoral immune response (antigen presentation pathway), intracellular and second messenger signaling (EIF2 signaling, regulation of EIF4 and p7056K signaling, PI3K/AKT signaling, Rho GDP-dissociation inhibitor (RhoGDI) signaling), neurotransmitters and other nervous systems (serotonin receptor signaling, regulation of actin-based motility by Rho, dopamine receptor signaling, gamma-aminobutyric acid (GABA) receptor signaling), organismal growth and development (clathrin-mediated endocytosis signaling), and pathogen-influenced signaling (clathrin-mediated endocytosis signaling, mechanisms of viral exit from host cells) (Table S18).

Our gene ontology analysis also identified 11 networks of genes with a score of 34 and containing 35 out of 35 focus molecules from out dataset. These top networks included gene networks associated with: 1) cancer, cell death, cellular assembly and organization built around signal transducer and activator of transcription 3 (*STAT3*); 2) free radical scavenging, small molecule biochemistry, and genetic disorder focused around nuclear factor of kappa light polypeptide gene enhancer in B-cells inhibitor, alpha (*NFKBIA*); 3) cellular movement, cancer, and gastrointestinal disease focused around v-rel reticulendotheliosis viral oncogene homolog A (*RELA*), which is also known as transcription factor p65 and binds to *NFKB1* or *NFKB2* to form the NF-kB complex; 4) nucleic acid metabolism, small molecule biochemistry, DNA replication, recombination, and repair built around amyloid beta (A4) precursor protein (*APP*), an integral membrane protein implicated in ion transport; 5) cell death, infectious disease, and carbohydrate metabolism focused around cyclin D1 (*CCND1*); 6) developmental disorder, genetic disorder, and hematological disease constructed around catenin (cadherin-associated protein), beta 1, 88 kDa (*CTNNB1*); 7) developmental disorder, genetic disorder, and metabolic disease built around v-erb-b2 erythroblastic leukemia viral oncogene homolog 2, neuro/gliobastoma derived oncogene homolog (*ERBB2*); 8) small molecule biochemistry, developmental disorder, and genetic disorder built around heme oxygenase (decycling) 1 (*HMOX1*); 9) kidney failure, renal and urological disease, and carbohydrate metabolism, which isn't focused around any one molecule; 10) molecular transport, free radical scavenging, and amino acid metabolism built around BCL2-like 1 (*BCL2L1*); 11) lipid metabolism, small molecule biochemistry, and molecular transport built around apolipoprotein E (*APOE*) (Table S19).

## Discussion

We used high-throughput RNA-Seq to characterize the baboon transcriptome using kidney biopsy samples collected from 14 animals of different sex and ranging in age from 6 to 23 years. Because the draft version of the baboon genome is not yet annotated, the human hg18 genome assembly was used to annotate genomic regions transcribed in the baboon kidney using a reference analysis as well as a RABT assembly approach. The high percentage of n's (up to 95.84%) within some of the baboon sequence reads likely resulted from using the human genome for gene annotation. Although similar, there are significant sequence differences between the two species, especially in the 5′ and 3′ UTRs. When aligned with the corresponding human genes, it appeared that most of the n's within the baboon sequences aligned with the UTRs or represented exons that are likely present in the baboon but not the human or vice versa.

Mapping the sequence reads to the human hg18 genome assembly enabled the identification of 20,714 coding RNAs and 1,118 hypothetical proteins or open reading frames using the reference analysis as well as an additional 10,430 coding RNAs and 678 hypothetical proteins or open reading frames, which are considered to be unique splice variants compared with the human hg18 reference genome, using the RABT assembly method. Moreover, annotation of transcripts based on their genomic coordinates within the hg19 genome assembly identified 350 coding RNAs and 6 hypothetical proteins or open reading frames unique to the hg18 genome assembly. Further analysis of our sequencing dataset using the latest genome assemblies for chimpanzee, orangutan, rhesus, and rat also identified 319 coding RNA variants and 4 hypothetical proteins in the baboon genome that appeared to be absent in the human hg18 and hg19 genome assemblies. The majority of these transcripts (294 out of 319) were identified using the rat genome assembly, which is likely due to the prevalent use of the rat model in scientific studies and better annotation of the rat genome.

Although we used a sample preparation method to enrich for mRNA from each total RNA sample, analysis of our sequencing dataset revealed numerous noncoding RNAs. Mapping the sequence reads to the human hg18 genome assembly enabled the identification of 1,492 noncoding RNAs (including 24 lincRNAs, 8 pre-miRNAs, and 31 snoRNAs) using the reference analysis as well as an additional 719 noncoding RNAs (including 22 lincRNAs, 28 pre-miRNAs, and 9 snoRNAs) using the RABT assembly method. Moreover, annotation of transcripts based on their genomic coordinates within the hg19 genome assembly identified 70 noncoding RNAs (including 1 lincRNA, 3 pre-miRNAs, and 1 snoRNA) unique to the hg18 genome assembly. No additional noncoding RNAs were identified using the latest genome assemblies for chimpanzee, orangutan, rhesus, and rat. Recent studies have shown that many noncoding RNAs function at the RNA level to affect the expression of neighboring protein-coding genes [60]–[63]. For example, miRNAs are 21- to 25-nucleotide-long RNAs that can either induce the degradation or suppress the translation of mRNAs, depending on whether they match their targets perfectly or approximately [64]. miRNAs have been implicated in the regulation of multiple biological and disease processes, including maintenance of glomerular homeostasis and progression of renal disease or injury [65]–[67]. Scientists have found that some noncoding RNAs can serve as biomarkers or potential therapeutic targets [68]–[70]. In a recent publication on the systems biology of kidney diseases [59], the authors discuss the potential advantages of miRNAs as disease biomarkers over standard mRNA or other protein-based profiles due to their stability in tissues and biological fluids, including urine [71], their protection from endogenous RNase degradation [71], and the tissue-specific nature of miRNA expression [72]. All of the noncoding RNAs identified in this study are novel for the baboon and will help scientists better understand their biological roles in human health and disease.

Altogether, analysis of our high-throughput sequencing dataset revealed the presence of 35,900 transcripts in the baboon kidney, which represented 15,669 genic genes based on common gene identifiers. Of the 15,669 genes identified, 6,124 were represented by only 1 transcript and 9,545 genes were represented by 2–61 transcript variants, with an average of 2.3 variants per gene. The number of expressed genes identified in this study is in relatively good agreement with previous findings of Ramsköld and colleagues [43] who recently used RNA-Seq to compare transcriptome composition across 5 cell lines and 11 tissues from human and mouse. The results of their study showed that the number of genes expressed in a tissue ranged from 11,199 to 15,518 genes with approximately 8,000 (∼75%) protein-coding genes ubiquitously expressed across all tissues and cell lines. Therefore, the majority of transcripts identified in our analysis of the kidney are presumed to be ubiquitously expressed genes and provide information on the core transcriptome of the baboon. Gene ontology analysis of our dataset revealed a large number of significant canonical pathways involved in metabolism and other core cellular functions, which also supports the findings of Ramsköld et al. that showed the ubiquitously expressed genes identified were mostly involved in these same molecular functions.

The largest number of transcripts and genes identified in the baboon kidney were located on chromosome 1 while the smallest number of transcripts and genes were located on the Y chromosome, which supports chromosomal distribution of genes in humans [73]–[75]. The lower number of transcripts identified on the Y chromosome is also likely due to the divergence between baboon and humans or other nonhuman primates. Although the genomes of human and chimpanzee, our closest living relative, differ in less than 1% of their DNA, Hughes and colleagues [75] recently noted that the Y chromosomes differ in 30% of their DNA content. This finding indicates that the Y chromosomes are changing at a much faster rate in both species than the rest of the genome. Therefore, the low number of transcripts we were able to identify on the Y chromosome of baboon is likely due to divergence in structure and gene content. Our data also revealed a larger number of genes, splice variants, and SNPs than average for chromosomes 17 and 19. Although these chromosomes are relatively small, previous findings have shown that chromosomes 19 and 17 are rich in protein-coding genes, having the highest and second highest gene density in the genome, respectively [73], [76], [77].

Most mammalian genes encode multiple isoforms with similar or diverse functions through the use of alternative promoters, polyadenylation sites, and splice sites. Unlike microarrays, RNA-Seq can identify differences in exon usage, alternative splicing, and allele-specific expression levels among samples to determine splice variant expression patterns to understand the function of a gene and how the function is altered in disease [43]–[49]. Recent studies using high-throughput sequencing of the human transcriptome have revealed much greater variability of the gene transcript isoforms than previously thought with approximately 40% of human genes producing five or more splice variants and up to 10% of them producing more than 10 alternate transcripts each [64], [78]–[81], which supports our finding of 2–61 transcript variants for 9,545 genes identified in the baboon kidney. This increase in transcriptome diversity plays a key role in regulating gene expression as different variants of a gene are expressed in different tissues or in the same tissue at different stages of development or in response to environmental changes and challenges [82]. Therefore, the identification of multiple variants from our sequencing dataset will help determine each gene's role in the kidney and identify the divergence of each gene between human and baboon.

Comparison of our dataset with the *Papio anubis* cDNAs contained in GenBank revealed the presence of 301 genes that had previously been identified in baboon. Alignment of the 750 transcript variants encoded by these 301 genes in our dataset with the baboon cDNA sequences from GenBank showed a high degree of similarity. Most notably were additional 5′- and 3′-UTR nucleotides in the baboon kidney cDNAs that were not included in the *Papio anubis* RefSeq cDNAs. Many stretches of n's in the baboon kidney cDNA sequences didn't align with baboon cDNA sequences from GenBank, which is likely due to the presence of exons in the human genome assembly but not the baboon. Excluding the 750 variants representing 301 genes already identified in *Papio anubis*, our dataset included 35,150 transcripts that are novel for the baboon. These 35,150 transcripts represented 15,369 genic genes based on common gene identifiers. The 15,369 genes included 6,018 genes represented by 1 variant and 9,351 genes represented by 2 to 61 variants, with an average of 2.3 variants per gene.

**Figure 1:**
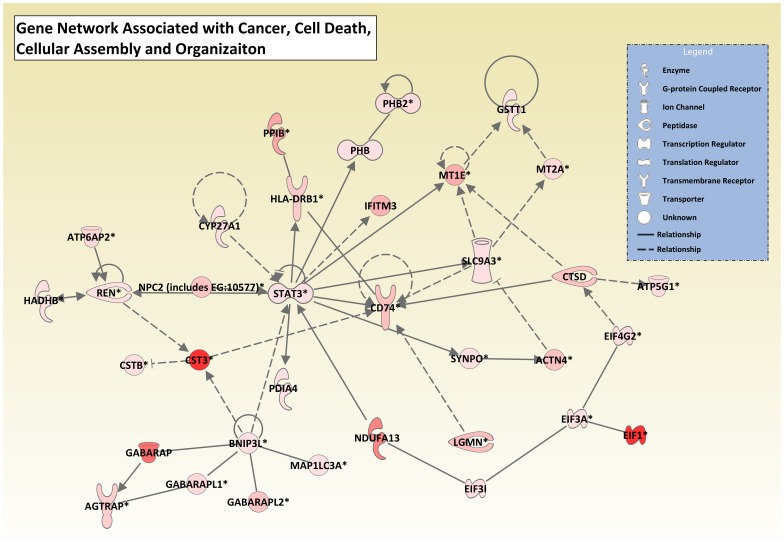
Graphical representation of oxidative phosphorylation and mitochondrial dysfunction pathways revealed significant genes in baboon kidney. IPA was used to identify canonical pathways from the IPA library that were most significant in the baboon kidney RNA-Seq dataset. Molecules from the dataset that met a fold coverage cutoff of 302.8 and were associated with a canonical pathway in Ingenuity's knowledge Base were considered for the analysis. Oxidative phosphorylation and mitochondrial dysfunction were identified as the two most significant pathways in the dataset. Groups of molecules in the oxidative phosphorylation and mitochondrial dysfunction pathways are represented as various shades of red. The intensity of the node color indicates the degree of fold coverage in the RNA-Seq dataset. Nodes shown in gray represent genes from the dataset that did not meet the fold coverage cutoff, and nodes shown in white represent genes that are in IPA's Knowledge Base but not in the dataset. Members within each significant group of molecules are shown in Table S20.

**Figure 2:**
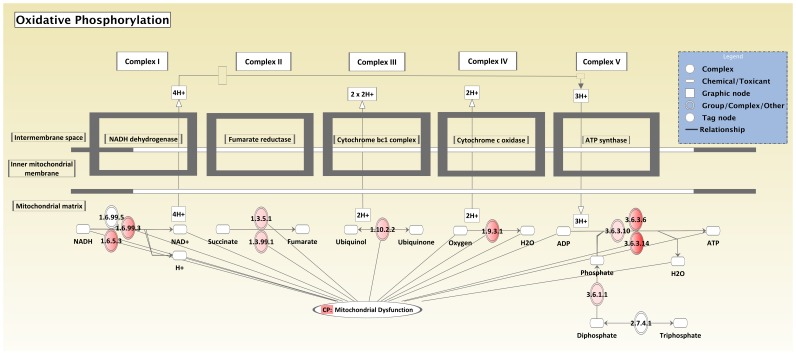
Network of genes associated with cancer, cell death, cellular assembly and organization in baboon kidney. Each transcript and corresponding fold coverage value was imported into IPA and mapped to its corresponding gene in the IPA Knowledge Base. A fold coverage value of 302.8 was set to limit the number of molecules considered for the analysis. Genes meeting the cutoff criteria were overlaid onto a global molecular network developed from information within the IPA Knowledge Base, and the networks were then algorithmically generated based on their connectivity. Graphical representation of the network reveals genes with highest fold coverage in the baboon kidney. Genes are represented as nodes of various shapes to represent the functional class of the gene product, and the biological relationship between two nodes is represented as a line. The intensity of the node color indicates the degree of fold coverage.

Gene ontology analysis of our dataset identified numerous biological functions and canonical pathways that were significant in the baboon kidney. The analysis revealed a diverse group of biological functions, including those associated with diseases or disorders and those associated with molecular and cellular functions. The analysis also identified a large number of significant metabolic pathways from our dataset that support known functions of the kidney, such as the metabolism of amino acids, carbohydrates, lipids, and vitamins [57], [58], [83]–[87]. In addition to these metabolic pathways, a number of signaling pathways were identified from the top 800 transcripts in our dataset. The most significant signaling pathways identified were oxidative phosphorylation and mitochondrial dysfunction (Figure 1 and Table S20), which are involved in many kidney diseases and other systemic diseases that induce oxidative stress in the kidney such as hypertension, diabetes mellitus, and hypercholesterolemia [88]–[90]. Furthermore, the hub molecules within the different *de novo* networks of genes identified in the baboon kidney are all known to play roles in kidney disease and/or function. For example, *STAT3* (shown in Figure 2) is a transcription factor that plays a role in many physiological processes, including the kidney's response to injury and the progression of certain renal diseases (e.g., diabetic nephropathy, renal fibrosis, and HIV-associated nephropathy) [91]. Previous findings have shown that *STAT3* plays an essential role in glucose homeostasis by regulating the expression of gluconeogenic genes in the liver [92]; therefore, it likely plays the same role in glucose homeostasis in the kidney [57]. *NFKB1*, which interacts with *RELA* to form the NF-kB complex, is essential for proper functioning of the immune system and modulates transepithelial sodium transport in the kidney [93], [94]. *APP* and *APOE* are involved in the transport of ions (e.g., sodium) and lipids, respectively. *CCND1* is involved in numerous cellular functions (e.g., growth, metabolism, and differentiation) and also functions as a proto-oncogene in renal cell carcinomas [95], [96]. Like *CCND1*, *CTNNB1* and *ERBB2* are also involved in numerous cellular functions and kidney disease [97], [98]. *HMOX1* is an essential enzyme involved in the regulation of renal vascular integrity and oxidative stress to reduce or prevent tissue injury in the kidney [99] and has been shown to attenuate the development of angiotensin II-dependent hypertension [100]. Lastly, numerous studies have shown that *BCL2L1* plays a key role in the regulation of apoptosis during renal cell injury [101]–[103].

In summary, we profiled the baboon transcriptome by sequencing the cDNA from kidney and mapping the obtained sequence reads to the human genome for the identification of known genes and novel transcribed regions independent of an existing annotated genome. We identified 35,900 RNAs, including 35,150 transcripts representing 15,369 genic genes that are considered to be novel for the baboon. Analysis of our RNA-Seq dataset also revealed 45,499 high-confidence SNPs and 29,813 InDels comparing baboon cDNA sequences with the human hg18 reference assembly. Gene ontology analysis identified numerous biological functions and canonical pathways that were significant in the baboon kidney, including a large number of metabolic pathways that support known functions of the kidney. Establishing the repertoire of genes expressed in the baboon kidney will establish a genomic resource for scientists using the baboon as an experimental model, will provide a resource for annotating the baboon genome sequence, and provide a better understanding for the molecular functions of the kidney.

## Materials and Methods

### Care and use of animals

All animal procedures were approved by the Institutional Animal Care and Use Committee at Texas Biomedical Research Institute (Texas Biomed) and conducted in facilities approved by the Association for Assessment and Accreditation of Laboratory Animal Care. The 14 baboons (*Papio hamadryas*) used in this study were chosen from seven different family groups housed in indoor/outdoor enclosures at the Southwest National Primate Research Center (SNPRC) at Texas Biomed and cared for in accordance with the U.S. Public Health Service Guide for the Care and Use of Laboratory Animals and the U.S. Animal Welfare Act. They were provided with a very active environmental enrichment program and trained to reduce stress and accomplish animal husbandry procedures cooperatively.

Kidney biopsies were obtained from 10 male and 4 female baboons ranging in age from 6 to 23 years. Eight kidney biopsies were collected from younger animals (age 6 to 8 years) while under anesthetic. These younger animals were fed twice daily using standard monkey chow supplemented with sodium and potassium (150 mmol sodium/500 g consumed and 90 mmol potassium/500 g), and water was provided *ad libitum*. The remaining 6 samples were obtained from older animals (age 9 to 23 years) at necropsy. These older animals were fed standard monkey chow twice daily, and water was provided *ad libitum*. It should be noted that these older animals were sacrificed for humane reasons due to poor health conditions (e.g., stomach hairball resulting in severe weight loss and dehydration, histoplasmosis, and old age) using Euthasol® Euthanasia Solution (Virbac Animal Health, Inc., Fort Worth, TX) or Beuthanasia®-D Special Euthanasia Solution (Schering-Plough Animal Health Corp., Union, NJ) administered in the right cephalic vein. Each tissue sample was collected, immediately frozen in liquid nitrogen, and stored at −80°C until use.

### RNA Isolation

Total RNA was isolated from each tissue using TRIzol Reagent (Invitrogen, Carlsbad, CA) according to the manufacturer's instructions. In brief, the tissue was homogenized in 1 ml of TRIzol Reagent per 50–100 mg of tissue using a PowerGen handheld homogenizer (Fisher Scientific, Pittsburgh, PA) and incubated for 5 min at 25°C to permit complete dissociation of nucleoprotein complexes. Chloroform (0.2 ml per 1 ml of TRIzol Reagent) was added to each sample, and the sample was shaken vigorously for 15 sec followed by a 3 min incubation at 25°C. The sample was centrifuged at 12,000×g for 15 min at 4°C, and the aqueous phase was transferred to a fresh tube. After chloroform extraction of the RNA, the aqueous phase was cleaned using Qiagen's RNeasy MinElute kit (Valencia, CA) according to manufacturer's instructions. The quantity and quality of each RNA extract was then assessed spectrophotometrically using a NanoDrop™ 8000 (Thermo Fisher Scientific, Wilmington, DE). RNA integrity was also confirmed by electrophoresis in a denaturing agarose gel, and extracts were stored at −80°C until use.

### Kidney transcriptome sequencing

The RNA extracts were processed for RNA-Seq using Illumina's mRNA-Seq Sample Preparation Kit according to the manufacturer's protocol. In brief, mRNA was purified from each RNA sample using poly-A selection, chemically fragmented into small pieces, and copied into first strand cDNA using random hexamer priming. Second strand cDNA synthesis was carried out using DNA Polymerase I and RNase H. Each cDNA library was then hybridized to an individual lane of a flow cell for cluster generation using the Illumina Paired-End Cluster Generation Kit v4 and Cluster Station and subsequently sequenced using the Illumina v4 Sequencing Kit and GAIIx Sequencer using a 101-cycle paired-end sequencing run.

### Sequence Read Assembly and Annotation

The sequencing output files (compressed FASTQ files, deposited in GenBank under accession # SRA053493) were analyzed using the free, open-source Tuxedo Suite comprising Bowtie, TopHat, and Cufflinks [49], [104], [105] as well as SAMtools [106] and the Genome Analysis Toolkit (GATK; [107]). In brief, paired sequence reads for each sample were aligned to the human genome (March 2006 (NCBI36/hg18)) using TopHat v 1.2.0 [105], which utilized Bowtie 0.12.7 for read mapping, to generate a set of putative exon-exon splice junction sites. The human gene annotations used in the Cufflinks analysis were from the University of California, Santa Cruz (UCSC) RefSeq hg18 refGene table (http://genome.ucsc.edu/cgi-bin/hgTables?hgsid=307358919&clade=mammal&org=Human&db=hg18&hgta_group=genes&hgta_track=refGene&hgta_table=0&hgta_regionType=genome&position=chrX%3A151073054-151383976&hgta_outputType=primaryTable&hgta_outFileName=), UCSC hg18 AceView genes table (http://genome.ucsc.edu/cgi-bin/hgTables?hgsid=307359873&clade=mammal&org=Human&db=hg18&hgta_group=genes&hgta_track=acembly&hgta_table=0&hgta_regionType=genome&position=chrX%3A151073054-151383976&hgta_outputType=primaryTable&hgta_outFileName=), and UCSC hg18 rnaGene table (http://genome.ucsc.edu/cgi-bin/hgTables?hgsid=307358919&clade=mammal&org=Human&db=hg18&hgta_group=genes&hgta_track=rnaGene&hgta_table=0&hgta_regionType=genome&position=chrX%3A151073054-151383976&hgta_outputType=primaryTable&hgta_outFileName=). The selected options allowed for up to 2 mismatches per 25-bp read segment to enable indel reporting and utilize a coverage search algorithm for greater sensitivity. The alignment of reads and read segments to the resulting Tophat splice junctions was used for transcript assembly.

GATK was used on the TopHat alignments for removal of PCR duplicates, indel realignment, and base quality score recalibration followed by SNP discovery and genotyping with exome-specific filtering [108]. Variant call output from the GATK Unified Genotyper was used to generate a consensus baboon genomic sequence with the human genome as a guide. SNPs from this analysis included polymorphisms between the human reference genome and the baboon kidney sequences. All confident bases were emitted and filtered on base quality and read depth. Nucleotides with a Phred-like base quality score greater than or equal to 17 and read depth greater than or equal to 4 were reported with an uppercase designation. Lowercase bases were used to indicate nucleotides with a quality score less than 17 and/or read depth less than 4. SNP discovery was then repeated over the consensus baboon kidney sequence. These SNPs were polymorphisms among the 14 baboon kidney samples. In addition to the quality checks performed using Bowtie and GATK, FastQC (version 0.9.6; http://www.bioinformatics.babraham.ac.uk/projects/fastqc/) was run on the 14 individual BAM files as well as the combined BAM file used for consensus sequence build and SNP calling to detect possible primer contamination and check per base sequence quality.

While the reference analysis identified transcripts that were structurally compatible with any reference transcript in the hg18 genome assembly, the Cufflinks v 0.9.3 RABT assembly method was also used on the TopHat alignments to construct, identify, and estimate expression of both known human and novel transcripts. Multi-read correction and upper quartile normalization options were enabled to increase accuracy and sensitivity, respectively. Differences in splicing, coding sequence output, and promoter usage among the 14 samples were identified with Cuffdiff [45], which calculated transcript expression levels using the Cufflinks transcript quantification engine. In brief, Cuffdiff used a merged GTF file of transcripts from all samples and Tophat alignment results as input to produce an output file containing expression levels of transcripts that included primary transcripts and genes. Cuffdiff also tracked the relative abundance of transcripts, taking into account the transcription start site and the relative abundances of primary transcripts for each gene. Tracking abundance of primary transcripts of a gene allowed validation of observed splicing events. Assembled transcript sequences were then generated with the gffread utility from the resulting baboon consensus sequence. Transcripts were annotated based on alignment of human genes and noncoding RNAs (http://cufflinks.cbcb.umd.edu).

### Annotation of transcripts from the RABT assembly that did not align with annotated genes in hg18 assembly

Transcript sequences from the RABT assembly with ≥1X coverage that did not align with annotated genes in the hg18 assembly were annotated based on their genomic coordinates within the hg19 human genome assembly (February 2009 (GRCh3/hg19)). The genomic coordinates (chromosome, chromosome start, chromosome end, and strand) for each of the transcripts were translated from the hg18 human genome assembly to the hg19 human genome assembly using the LiftOver utility in Galaxy (http://g2.bx.psu.edu) with a 0.95 minimum ratio of remapped bases. Galaxy was then used to join the translated coordinates with the human hg19 gene track from the Table Browser in the UCSC Genome Browser (http://genome.ucsc.edu) based on genomic interval overlap, using 1 bp as the minimum required overlap between the 2 gene tracks.

Transcript sequences from the RABT assembly that were not annotated using the hg19 gene track were then translated and mapped to the chimpanzee (March 2006 (CGSC 2.1/panTro2)), orangutan (July 2007 (WUGSC 2.0.2/ponAbe2)), rhesus (January 2006 (MGSC Merged 1.0/rheMac2)), and rat (November 2004 (Baylor 3.4/rn4)) genomes based on genomic interval overlap, using 1 bp as the minimum required overlap between the gene tracks.

### Comparison of RNA-Seq Transcripts to GenBank Papio Genes

Transcripts identified from the reference and RABT assemblies were compared with *Papio* NCBI RNA reference sequences (http://www.ncbi.nlm.nih.gov/nuccore). The MAFFT (Multiple Alignment using Fast Fourier Transform) tool from EMBL-EBI (European Molecular Biology Laboratory-European Bioinformatics Institute; (http://www.ebi.ac.uk/Tools/msa/mafft/) was used to compare the coding region of 108 baboon kidney RNA-Seq cDNA sequences with the *Papio* sequences in GenBank.

### Pathway Analysis

Coverage values for all transcripts identified from the reference and RABT assemblies were imported into IPA to identify the significant biological functions, canonical pathways, and networks of genes in the baboon kidney transcriptome. Biological functions are categories into which genes are classified based on their cellular or physiological role in an organism. A canonical pathway is a well-established signaling or metabolic pathway that is manually curated on the basis of published literature. Canonical pathways are fixed prior to data input. Networks are distinct from canonical pathways in that they are built *de novo* from input data based on known molecular interactions identified in the published scientific literature.

To identify biological functions, canonical pathways, and gene networks that were most significant in our dataset, the top 800 molecules that were associated with a canonical pathway in Ingenuity's Knowledge Base were considered for analysis. The significance of the association between the dataset and the canonical pathway was measured in 2 ways: 1) A ratio of the number of molecules from the dataset that mapped to the pathway divided by the total number of molecules that mapped to the canonical pathway was displayed. 2) Fisher's exact test was used to calculate a *p*-value determining the probability that the association between the genes in the dataset and the canonical pathway was explained by chance alone. To determine networks of genes significantly affected in the baboon kidney, molecules were overlaid onto a global molecular network developed from information contained in Ingenuity's Knowledge Base. Networks of molecules were then algorithmically generated based on their connectivity. The functional analysis of a network identified the biological functions and/or diseases that were most significant to the molecules in the network. The network molecules associated with biological functions and/or diseases in the Knowledge Base were considered for the analysis. Right-tailed Fisher's exact test was used to calculate a *p*-value determining the probability that each biological function and/or disease assigned to that network is due to chance alone.

## Supporting Information

Table S1 Coding RNA transcripts identified using human hg18 reference analysis of sequencing reads. This table includes: Homo sapiens NCBI RefSeq ID, gene ID, gene description, transcript length, number of N's, percent N's, fold coverage, FPKM, coding sequence region (CDS), chromosome, chromosome start, chromosome end, strand, exons, and FASTA consensus sequence (baboon). (FASTQ sequences are deposited in GenBank, Accession # SRA053493).
(XLSX)

Table S2 Hypothetical proteins and open reading frames identified using human hg18 reference analysis of sequencing reads. This table includes: Homo sapiens NCBI RefSeq ID, gene ID, gene description, transcript length, number of N's, percent N's, fold coverage, FPKM, CDS, chromosome, chromosome start, chromosome end, strand, exons, and FASTA consensus sequence (baboon). (FASTQ sequences are deposited in GenBank, Accession # SRA053493).
(XLSX)

Table S3 Splice variant count of coding RNAs identified using human hg18 reference analysis of sequencing reads. This table includes gene ID and number of variants per gene.
(XLSX)

Table S4 Noncoding RNA transcripts identified using human hg18 reference analysis of sequencing reads. This table includes: Homo sapiens NCBI RefSeq ID, gene ID, gene description, transcript length, number of N's, percent N's, fold coverage, FPKM, chromosome, chromosome start, chromosome end, strand, exons, and FASTA consensus sequence (baboon). (FASTQ sequences are deposited in GenBank, Accession # SRA053493).
(XLSX)

Table S5 Splice variant count of noncoding RNAs identified using human hg18 reference analysis of sequencing reads. This table includes gene ID and number of variants per gene.
(XLSX)

Table S6 Coding RNA transcripts identified using RABT assembly of sequencing reads. This table includes: Cufflinks tracking ID, class code (“ = ” means sequence has a complete match of intron chain compared with reference transcript, “j” means sequence is a potentially novel isoform (fragment) with at least one splice junction shared with the reference transcript, “o” means sequence has a generic exonic overlap with the reference transcript, “x” means sequence has exonic overlap with the reference transcript on the opposite strand, and “s” means an intron of the transfrag overlaps the reference intron on the opposite strand (possibly due to read mapping errors)), nearest Homo sapiens NCBI RefSeq ID, gene ID, gene description, XLOC (a unique internal identifier for the locus being tested), TSS ID (an identifier for the transcript's inferred start site that determines which primary transcript the processed transcript is believed to come from), CDS, transcript length, number of N's, percent N's, fold coverage, FPKM, chromosome, chromosome start, chromosome end, strand, exons, and FASTA consensus sequence (baboon). (FASTQ sequences are deposited in GenBank, Accession # SRA053493).
(XLSX)

Table S7 Hypothetical proteins and open reading frames identified using RABT assembly of sequencing reads. This table includes: Cufflinks tracking ID, class code, nearest Homo sapiens NCBI RefSeq ID, gene ID, gene description, XLOC, TSS ID, CDS, transcript length, number of N's, percent N's, fold coverage, FPKM, chromosome, chromosome start, chromosome end, strand, exons, and FASTA consensus sequence (baboon). (FASTQ sequences are deposited in GenBank, Accession # SRA053493).
(XLSX)

Table S8 Noncoding RNA transcripts identified using RABT assembly of sequencing reads. This table includes: Cufflinks tracking ID, class code, nearest Homo sapiens NCBI RefSeq ID, gene ID, gene description, XLOC, TSS ID, transcript length, number of N's, percent N's, fold coverage, FPKM, chromosome, chromosome start, chromosome end, strand, exons, and FASTA consensus sequence (baboon). (FASTQ sequences are deposited in GenBank, Accession # SRA053493).
(XLSX)

Table S9 Baboon RNAs identified using RABT assembly of sequences and annotated using human hg19 assembly. This table includes: Cufflinks tracking ID, class code, XLOC, TSS ID, transcript length, number of N's, percent N's, fold coverage, FPKM, hg18 chromosome, hg18 chromosome start, hg18 chromosome end, hg18 strand, hg18 exons, gene ID, gene description, hg19 NCBI RefSeq ID, hg19 chromosome, hg19 chromosome start, hg19 chromosome end, hg19 strand, hg19 start codon, hg19 stop codon, hg19 number of exons, hg19 exon lengths, hg19 exon start positions relative to chromosome start, and FASTA consensus sequence (baboon). (FASTQ sequences are deposited in GenBank, Accession # SRA053493).
(XLSX)

Table S10 Splice variant count of novel baboon coding RNAs compared with human hg18 reference genome. This table includes gene ID and number of variants per gene.
(XLSX)

Table S11 Splice variant count of novel baboon noncoding RNAs compared with human hg18 reference genome. This table includes gene ID and number of variants per gene.
(XLSX)

Table S12 Baboon RNAs identified using RABT sequence assembly and annotated using ponAbe2 and rheMac2 genomes. This table includes: Cufflinks tracking ID, class code, XLOC, TSS ID, transcript length, number of N's, percent N's, fold coverage, FPKM, hg18 chromosome, hg18 chromosome start, hg18 chromosome end, hg18 strand, hg18 exons, gene ID, gene description, ponAbe2/rheMac2 NCBI RefSeq ID, ponAbe2/rheMac2 chromosome, ponAbe2/rheMac2 chromosome start, ponAbe2/rheMac2 chromosome end, ponAbe2/rheMac2 strand, ponAbe2/rheMac2 start codon, ponAbe2/rheMac2 stop codon, ponAbe2/rheMac2 number of exons, ponAbe2/rheMac2 exon lengths, ponAbe2/rheMac2 exon start positions relative to chromosome start, and FASTA consensus sequence (baboon). (FASTQ sequences are deposited in GenBank, Accession # SRA053493).
(XLSX)

Table S13 Baboon RNAs identified using RABT sequence assembly and annotated using rn4 genome assembly. This table includes: Cufflinks tracking ID, class code, XLOC, TSS ID, transcript length, number of N's, percent N's, fold coverage, FPKM, hg18 chromosome, hg18 chromosome start, hg18 chromosome end, hg18 strand, hg18 exons, gene ID, gene description, rn4 NCBI RefSeq ID, rn4 chromosome, rn4 chromosome start, rn4 chromosome end, rn4 strand, rn4 start codon, rn4 stop codon, rn4 number of exons, rn4 exon lengths, rn4 exon start positions relative to chromosome start, and FASTA consensus sequence (baboon). (FASTQ sequences are deposited in GenBank, Accession # SRA053493).
(XLSX)

Table S14 Splice variant count of novel baboon RNAs compared with human hg18 and hg19 reference genomes. This table includes gene ID and number of variants per gene.
(XLSX)

Table S15 Splice variant count of all baboon RNAs identified from RNA-Seq dataset. This table includes gene ID and number of variants per gene for 35,900 transcripts identified in RNA-Seq dataset, 750 transcripts identified in GenBank, and 35,150 transcripts novel for baboon.
(XLSX)

Table S16 High-confidence SNPs identified among the 14 baboon kidney transcriptome samples. This table includes: chromosome, position, reference nucleotide, alternate nucleotide, quality score, NCBI RefSeq ID, gene ID, region, amino acid substitution, reference amino acid, and alternate amino acid.
(XLSX)

Table S17 Significant biological functions identified from top 800 transcripts in baboon kidney RNA-Seq dataset. This table includes the p-values and molecules for each biological function from the IPA Knowledge Base identified as being significant in the baboon kidney dataset.
(XLSX)

Table S18 Significant canonical pathways identified from top 800 transcripts in baboon kidney RNA-Seq dataset. This table includes the –log(p-value), ratio, and molecules for each canonical pathway from the IPA Knowledge Base identified as being significant in the baboon kidney dataset.
(XLSX)

Table S19 Top biological gene networks identified from top 800 transcripts in baboon kidney RNA-Seq dataset. This table includes the score (displayed as the –log(p-value) and indicating the likelihood that the assembly of focus genes in a network could be explained by random chance alone), focus molecules, and top biological functions associated with each network.
(XLSX)

Table S20 Members of each significant group of molecules within the oxidative phosphorylation and mitochondrial dysfunction pathways. This table includes members within each group of molecules represented as various shades of red. The intensity of the node color indicates the degree of fold coverage in the RNA-Seq dataset. Nodes shown in gray represent genes from the dataset that did not meet the fold coverage cutoff, and nodes shown in white represent genes that are in IPA's Knowledge Base but not in the dataset.
(XLSX)
